# Research and development of focused proton microbeam irradiation system, SPICE for radio-biological studies.

**DOI:** 10.1093/jrr/rrt195

**Published:** 2014-03

**Authors:** Teruaki Konishi, Masakazu Oikawa, Noriyoshi Suya, Alisa Kobayashi, Takeshi Maeda, Yukio Uchihori, Yoshiyuki Shirakawa

**Affiliations:** 1 Research Development and Support Center, National Institute of Radiological Sciences, Japan; 2 Space Radiation Research Unit, International Open Laboratory, National Institute of Radiological Sciences, Japan

**Keywords:** space radiation, microbeam, bystander effects, low-dose effect

## Abstract

There is continuing interest for the use of microbeam irradiation systems designed to deliver a defined number of charged particles on a single cell with a resolution of a few micrometers. Irradiation of an exact number of charged particles on a single cell means that the limitations of the Poisson distribution of the number of charged particles can be overcome. Moreover, microbeams are particularly useful for the field of radiation-induced non-targeted effects, so-called bystander effects that are considered to be one of the major effects in the low-dose region. Thus, microbeam technique is one of the powerful tools for investigating studies related to radiation effect and risk of low dose in space radiation for astronauts and cosmonauts.

Our microbeam irradiation system, the Single-Particle Irradiation system to CEll (SPICE) provides a 3.4 MeV proton microbeam focused with a quadrupole magnetic lens on an upward vertical beam line. SPICE was severely damaged by the Tohoku-oki Earthquake on 11 March 2011, and was out of operation for about a year and a half. We have successfully reconstructed the facility, and it is now operational with system refinements. At present, SPICE is the only proton microbeam facility in Japan at which a single-ion single-cell irradiation can be performed on mammalian cells with stability and high throughput using an upward vertical beam of below 2-μm diameter, focused with a magnetic quadrupole triplet lens [ 1]. A variety of irradiation modes have been established for radiation-induced bystander effects, cytoplasm irradiation etc. An example of cells targeted with a multi-position targeting mode is shown in Fig. 1, which cells were targeted with five different positions in the nucleus with 50 protons per position. SPICE has been administrated as a ‘Joint-use facility for Collaborative Research’, and thus researchers outside NIRS can apply for beam time of SPICE after their research proposals are approved.

**Fig. 1.**
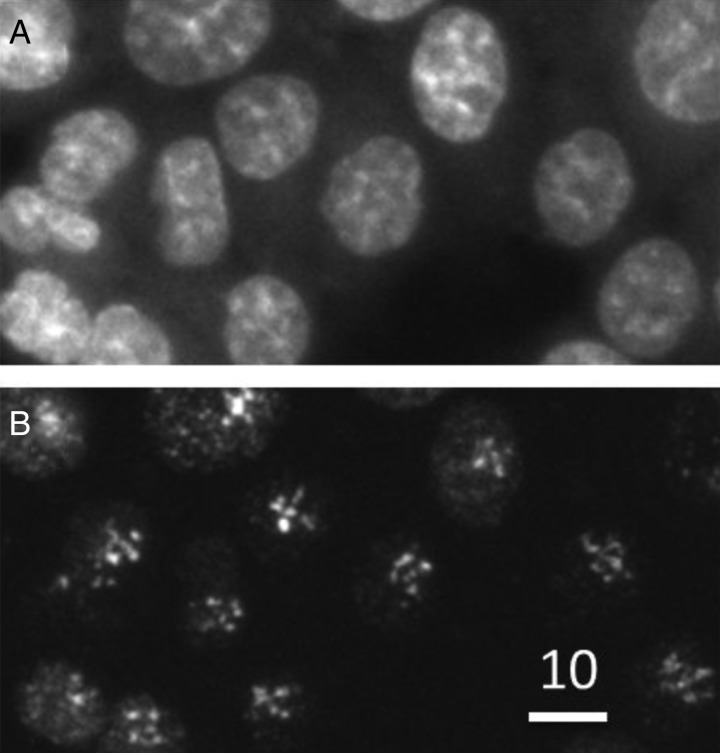
(**A**) Nuclei of HCT116 cells were targeted with five different positions (3-µm apart), and 50 protons were delivered to each position. (**B**) Cells were incubated for 1 h and then fixed for immunostaining against γ-H2AX.

(**A**) Nuclei of HCT116 cells were targeted with five different positions (3-µm apart), and 50 protons were delivered to each position. (**B**) Cells were incubated for 1 h and then fixed for immunostaining against γ-H2AX.
